# COVID-19-Induced Complete Heart Block: Case Series and Literature Review

**DOI:** 10.7759/cureus.37517

**Published:** 2023-04-13

**Authors:** Raghav Bassi, Zeeshan Ismail, Joshua K Salabei, Kipson Charles, Asad A Haider, Abdullahi Hussein, Andrew Smock

**Affiliations:** 1 Internal Medicine, University of Central Florida College of Medicine, Graduate Medical Education/North Florida Regional Medical Center, Gainesville, USA; 2 Cardiology, University of Central Florida College of Medicine, Graduate Medical Education/North Florida Regional Medical Center, Gainesville, USA

**Keywords:** covid-induced myocarditis, covid-19-induced arrhythmia, arrhythmia, complete heart block, heart block, coronavirus disease 2019 (covid-19)

## Abstract

The severe acute respiratory syndrome coronavirus 2 (SARS-CoV-2) pandemic has led to the emergence of a wide range of complications, including those affecting the cardiovascular system. In this case series, we present four patients who developed complete atrioventricular block, a serious and potentially life-threatening heart rhythm disorder, during the course of their coronavirus disease 2019 (COVID-19) illness. The mechanisms by which SARS-CoV-2 may lead to arrhythmias are not fully understood but may involve direct infection and damage to heart tissue, as well as inflammation and cytokine storms. The extent and duration of complete heart block varied among these cases, highlighting the need for further research to understand the spectrum of disease and to improve mortality and morbidity in future waves of SARS-CoV-2 infections. We hope that this case series will draw attention to this serious complication of COVID-19 and inspire further research to improve management and outcomes for affected patients.

## Introduction

It has been several years since the first case of severe acute respiratory syndrome coronavirus 2 (SARS-CoV-2) was discovered in Wuhan, China. However, clinically SARS-CoV-2 continues to challenge clinicians. Although the virus primarily affects the respiratory system, it is known to involve other organ systems, such as the cardiac, hematologic, and renal systems. Cardiac arrhythmias are frequently seen in severe coronavirus disease 2019 (COVID-19). Some studies have cited arrhythmias occurring in up to 44% of patients and bradycardia (sinus bradycardia and AV block) occurring in up to 24.9% of patients [1,2]. Although the etiology for arrhythmias in COVID-19 has not been established, it is hypothesized that a combination of a pro-inflammatory state, cytokine storm, viral myocyte infiltration, hypoxemia, and fulminant myocarditis disrupts the cardiac conduction system [1,2].

Furthermore, additional studies have postulated that these arrhythmias can occur due to electrolyte abnormalities, QT prolongation medications used in the therapy of COVID-19, autonomous inflammation, underlying cardiovascular comorbidities, and hypoxia [3]. The underlying pathophysiology is thought to be due to underlying viral myocarditis resulting in myocardial inflammation with severe necrosis. This can ultimately lead to re-entry points in the electrical circuit, evolving into ventricular tachycardia and ventricular fibrillation. Furthermore, known sequelae of COVID-19’s severe inflammatory phase include the development of hypoxia which can contribute to arrhythmias. Arrhythmias (transient atrioventricular {AV} block) among critically ill patients have been previously associated with underlying myocarditis or increased pulmonary artery pressure [4].

## Case presentation

Case 1

A 56-year-old female with a pertinent past medical history (PMH) of type 2 diabetes mellitus, hypertension, obesity class 3, and hyperlipidemia was transferred from an outside medical facility for an extracorporeal membrane oxygenation (ECMO) evaluation. She presented to the previous facility with a one-week duration of shortness of breath and tested positive for SARS-CoV-2. She was started on standard medical therapy including remdesivir, dexamethasone, therapeutic enoxaparin, and supplemental oxygen. She subsequently developed acute hypoxic respiratory failure requiring intubation and was transferred to our facility two days later. A transthoracic echocardiogram (TTE) performed prior to transfer revealed a normal left ventricular ejection fraction (LVEF) of 55-60%, no wall motion abnormalities, normal right ventricular function, and a right ventricular systolic pressure (RVSP) of 25 mmHg.

**Figure 1:**
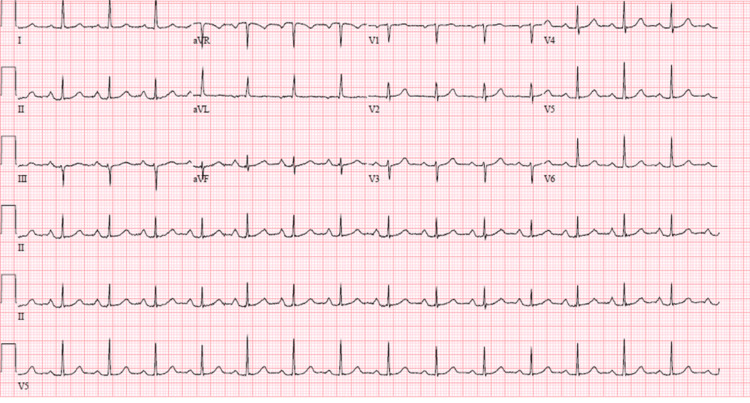
An EKG revealing sinus tachycardia without any ST or T wave abnormalities.

On arrival at our facility, she was intubated, sedated, and on mechanical ventilation. On physical examination, her heart rate was 105 beats per minute (bpm), blood pressure was 124/78 mmHg, and oxygen saturation of 94% on 100% oxygen. Other abnormal physical examination findings included mild rhonchi and 1+ peripheral pitting edema. Further objective data can be found in Table 1. Markedly elevated inflammatory markers (c-reactive protein {CRP}, erythrocyte sedimentation rate {ESR}, ferritin, lactate dehydrogenase {LDH}, d-dimer) were noted, along with a mild troponin elevation and vitamin D deficiency. Further workup included an electrocardiogram (EKG) which revealed normal sinus rhythm without T wave abnormality and a chest x-ray with the bilateral parenchymal infiltrative disease (Figure 1).

**Table 1:** Summary of labs and medications on admission and during heart block of all four cases. NP: not performed

Parameters	Case 1		Case 2		Case 3		Case 4		Reference
	Level on admission	During heart block	Level on admission	During heart block	Level on admission	During heart block	Level on admission	During heart block	Normal range
Days since first positive SARS-CoV-2 test	Day 6	Day 15	Day 8	Day 28	Day 8	Day 12	Day 1	Day 4	
White blood cell count	12.8	7	14.2	5.3	14	9	16	18	(4.5 - 11.0 thousand/mm^3^)
Hemoglobin	11	10	13	11.2	12.5	10	10	8	(12.0 - 15.0 g/dL)
Hematocrit	28	28.8	38.9	33.3	37.5	30.2	30	24	(35.0-49.0%)
Platelet count	348	48	419	208	200	180	140	90	150-450 thousand/mm^3^
Sodium	132	148	133	132	135	133	128	135	136-145 mmol/L
Potassium	3.5	4	3.6	3.7	2.8	3.6	3.6	3.6	3.5-5.1 mmol/L
Chloride	92	118	103	93	95	96	97	96	98-107 mmol/L
Carbon dioxide	34	22	23	33	24	21	20	18	21-31 mEq/L
Blood urea nitrogen	15	69	17	14	16	18	45	80	7-18 mg/dL
Creatinine	0.8	1.91	0.67	0.42	0.9	1.01	2	4.2	0.6-1.30 mg/dL
Magnesium	2	2.3	2.8	2.5	1.8	2	1.9	3.2	1.7-2.2 mmol/L
Phosphorous	4	2.3	4	3.8	2.3	3.2	2.5	2.8	2.5-4.29 mg/dL
Thyroid-stimulating hormone	2.2	2.3	2	1.2	1.8	NP	3.1	NP	0.5-5.0 mIU/L
Troponin	0.180	0.020	0.010	<0.010	<0.010	<0.010	0.870	0.040	0.00-0.045 ng/mL
Pro-BNP	52	NP	82	NP	NP	NP	3000	NP	0-900
D-dimer	0.523	2.23	0.48	10.68	3	3.4	0.4	NP	<0.5 mg/L
LDH	200	500	207	530	860	1500	565	NP	87-241 U/L
Ferritin	900	2000	421.7	737	800	850	1112	NP	20-250 ng/mL
CRP	12	>27	13	26.4	10	8	22	NP	<5 mg/L
ESR	60	83	NP	NP	10	NP	15	NP	0-29 mm/h
Vitamin D	17	NP	13	21	19	NP	10	NP	30-50 ng/mL
Lactic acid	1.6	1.7	1.9	1.4	1.2	NP	4	8	<2
Hemoglobin A1C	8.8	NP	4.4	NP	9.4	NP	6	NP	<5.7%
ABG	7.22/72/64/29.9	7.33/38/67/21.8	NP	7.4/45/71/23	7.4/45/71/23	NP	7.55/31/58/26.4	NP	7.4/40/60-80/24
Medications	Dexamethasone, remdesivir, convalescent plasma, acetaminophen, ceftriaxone, famotidine, aripiprazole, benztropine, atorvastatin	Dexmedetomidine, propofol, fentanyl, cefepime, bivalirudin, vecuronium, furosemide, pantoprazole, acetaminophen, glargine + lispro	Lisdexamfetamine, levothyroxine, buspirone, fluoxetine, seroquel, topiramate, oxycodone/paracetamol, dexamethasone, clindamycin, albuterol/ipratropium nebs	Dexmedetomidine, versed, fentanyl, ketamine, bivalirudin, vecuronium, cefepime, albuterol/ipratropium nebs, vecuronium, furosemide, famotidine, acetaminophen, hydralazine, fluoxetine, glargine + novolin	No home medications	Heparin, azithromycin, ceftriaxone, remdesivir, methylprednisolone, albuterol/ipratropium nebulizers senna/docusate, famotidine, glargine, lispro	Furosemide, spironolactone, propranolol, doxycycline, empagliflozin	Methylprednisolone, norepinephrine, albumin, ampicillin/sulbactam, cefepime, lispro, pantoprazole, sevelamer, senna, docusate	-

Given that she was in severe acute respiratory distress syndrome (ARDS), she was ventilated in a prone position. This initially improved her oxygenation; however, her disease continued to progress, prompting cardiothoracic surgery to initiate venovenous (VV) ECMO through the right internal jugular vein.

**Figure 2:**
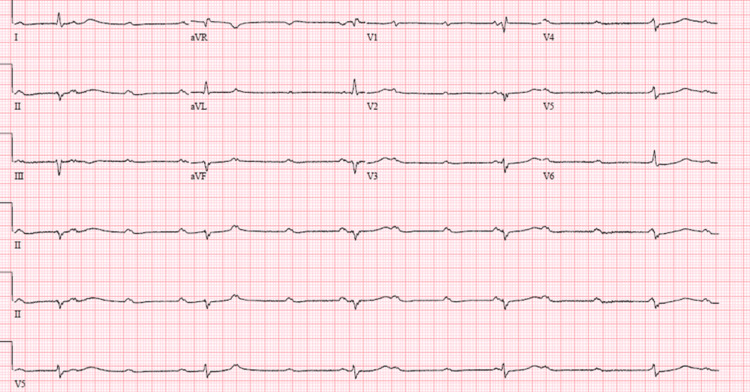
An EKG revealing a complete third-degree heart block.

On the 15th day after her initial positive SARS-CoV-2 test, she developed a pulseless electrical activity arrest, and cardiopulmonary resuscitation (CPR) was initiated. Return of spontaneous circulation was achieved after 30 seconds of CPR and one administration of epinephrine. A complete heart block was noted on the monitor, and transcutaneous pacing was initiated. Notable labs and medications at the time can be found in Table 1. There were no electrolyte disturbances during the time of the complete heart block. Due to the persistence of heart block, a temporary right femoral transvenous pacing device was then installed (Figure 2). A post-cardiac arrest TTE revealed a hyperdynamic left ventricle, a severely dilated right ventricle with reduced systolic function, and a trivial pericardial effusion. A contrasted tomography angiography pulmonary embolism scan (CTA PE) was not able to be completed due to the patient's severity of illness. She required significant pacing over the ensuing days, and despite optimal medical management, she developed multiorgan failure and expired nine days later.

Case 2

A 31-year-old white female with PMH of hypothyroidism and obesity class two presented with complaints of dyspnea on exertion and intermittent diarrhea. One week prior to the presentation, she tested positive for SARS-CoV-2, and her primary care provider began treatment with dexamethasone, amoxicillin/clavulanate, and home ipratropium bromide/albuterol. However, she continued to decline, prompting her to come to the hospital.

**Figure 3:**
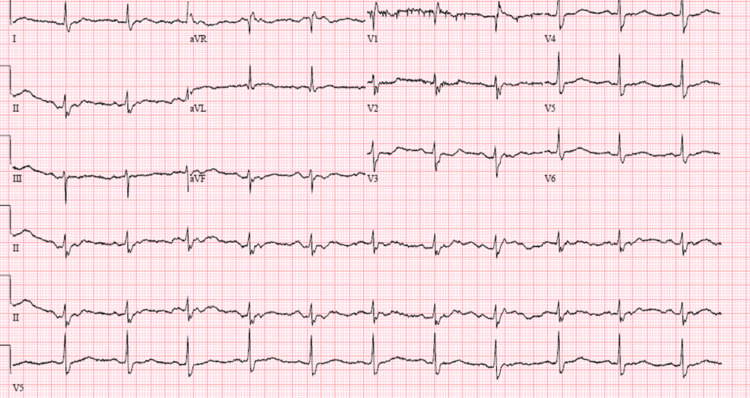
EKG revealed a normal sinus rhythm with an incomplete RBBB. RBBB: right bundle branch block

On presentation, oxygen saturation was 89% on a 7 L nasal cannula, and her other vitals and labs were within normal limits. Further objective data can be found in Table 1. A CTA PE scan identified diffuse pulmonary parenchymal ground-glass opacities with no evidence of a pulmonary embolism, and EKG exhibited normal sinus rhythm with an incomplete; right bundle branch block (RBBB) (Figure 3). TTE findings revealed an ejection fraction of 50-55%, no focal wall motion abnormalities, mild mitral regurgitation, normal RV function, with no effusions noted.

Pulse dose steroids, remdesivir, convalescent plasma, supplemental oxygen, and other COVID-19-appropriate vitamins were initiated per our hospital protocol at the time. Despite these measures, she continued to develop worsening acute hypoxic respiratory failure requiring intubation, paralyzation, proning, and inhaled epoprostenol. In spite of this escalation in care, she remained in severe ARDS as per the Berlin criteria, prompting the initiation of VV ECMO.

**Figure 4:**
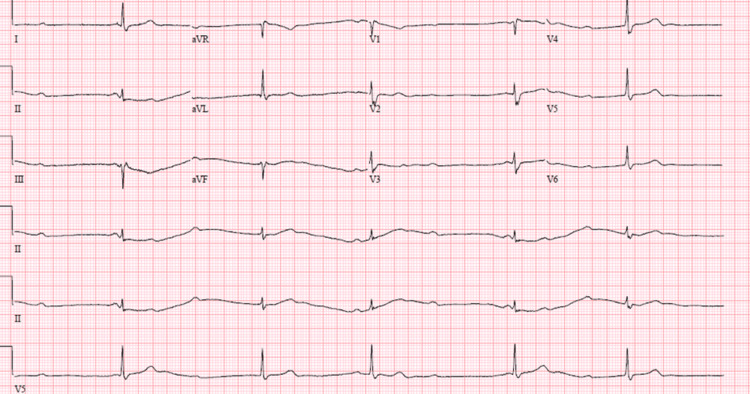
An EKG revealing a third-degree AV block.

On day 21 of hospitalization, she was noted to have a heart rate of 41 bpm, and a narrow complex complete heart block was noted on a 12-lead EKG (Figure 4). Electrolytes at the time of the heart block were all unremarkable. Transcutaneous pacing was initiated. She continued to require pacing intermittently, so a temporary transvenous pacing device was placed. A TTE revealed normal LV function; however, a dilated right ventricle (RV) was noted with severely reduced systolic function with an associated small pericardial effusion. A CTA PE was subsequently repeated which showed diffuse ground glass opacities bilaterally without evidence of pulmonary artery occlusion. Over the succeeding few days, her pacing requirements decreased, and after eight days, the transvenous pacing device was removed.

She then proceeded to have a long hospital course while slowly improving and eventually being discharged to a long-term care facility for further recovery. Prior to discharge, a single-chamber permanent pacemaker was placed. Outpatient follow-up approximately six months after discharge revealed that she remained in normal sinus rhythm, not requiring any pacing, and had recovered normal right and left heart systolic function.

Case 3

A 55-year-old white male presented with progressive shortness of breath, fevers, myalgias, and loss of taste and smell for two weeks. He had tested positive for COVID-19 eight days prior to the presentation. His symptoms had mostly resolved except for progressive shortness of breath that persisted, leading to his presentation to our hospital. He had no further pertinent past medical, surgical, or family history. He was an active smoker, reporting 11 pack-years of smoking, social alcohol drinker, and had no prior drug use except occasional marijuana. He did not take any medications at home and would take daily multivitamins.

**Figure 5:**
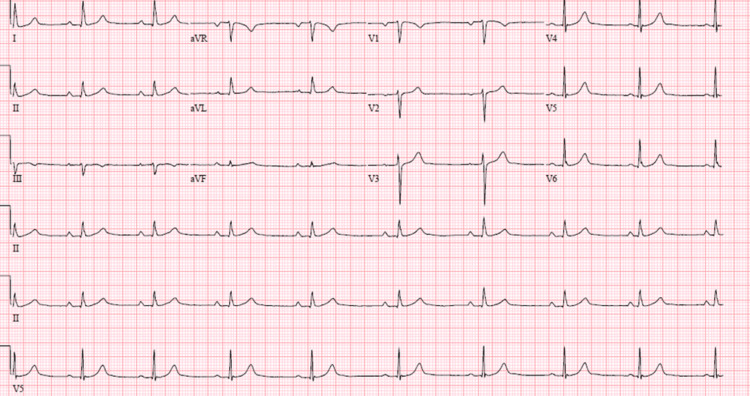
EKG revealed normal sinus rhythm without significant T wave abnormalities.

On physical examination, his heart rate was 105 bpm, his blood pressure was 115/75 mmHg, and his oxygen saturation was 92% on a 6 L nasal cannula. His cardiopulmonary examination was normal. Further laboratory findings can be found in Table 1. His EKG was consistent with sinus tachycardia and without T-wave abnormalities (Figure 5). CTA PE study revealed a left lower lobe segmental perfusion deficit and a TTE identified an RV thrombus roughly measuring 1.2 x 1.5 cm adhering to the chordae tendineae. A lower extremity ultrasound did not reveal any deep venous thromboses.

**Figure 6:**
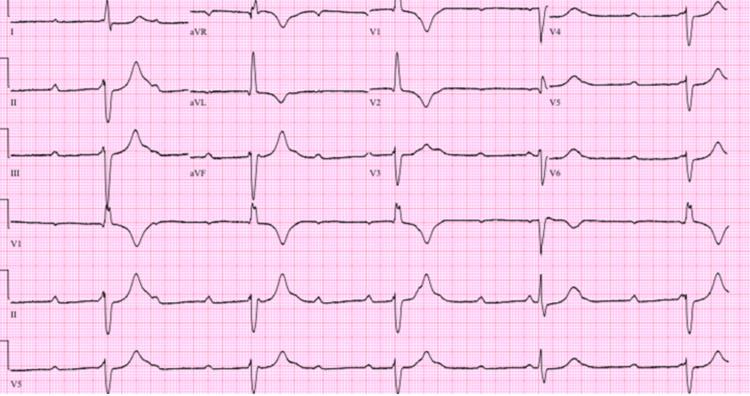
EKG revealed a third-degree heart block.

He was then started on unfractionated heparin, dexamethasone, remdesivir, and famotidine, and admitted to the intensive care unit (ICU). Over the next few days, his oxygen requirements improved. However, on day five of hospitalization, he was noted on telemetry to have an intermittent complete heart block and a heart rate ranging from 40-50 bpm (Figure 6). During these episodes, he remained hemodynamically stable without an increase in oxygen requirements or any obvious electrolyte disturbances. Transcutaneous pads were placed as per protocol. Subsequent tests including troponin and EKG were unchanged, and TTE re-demonstrated the right ventricular thrombus. The patient’s intermittent complete heart block persisted for about 24 hours but later spontaneously converted to normal sinus rhythm. He was monitored for several days in the ICU, and after 48 hours of normal sinus rhythm, he was then downgraded to the floor. There were no complications during his hospital stay and he was subsequently discharged home and instructed to follow-up outpatient with cardiology.

Case 4

A 62-year-old white male with a PMH of hypertension, morbid obesity, and chronic obstructive pulmonary disease presented with shortness of breath, abdominal fullness, and mild peripheral edema of two days duration. On presentation, he tested positive for COVID-19. Due to his acute respiratory distress, an arterial blood gas analysis was performed and it revealed an acute hypoxic and hypercapnic respiratory failure. He was placed on a bi-level non-invasive positive pressure ventilation machine (BiPAP) for respiratory support.

On further questioning, he had quit smoking five years ago; however, he had a total of 30 pack-years history of smoking. Also, he reported previous binge drinking in his 20s but no recent alcohol use. Surgical and family history were unremarkable. Home medications included spironolactone, furosemide, albuterol sulfate, and umeclidinium/vilanterol combination.

**Figure 7:**
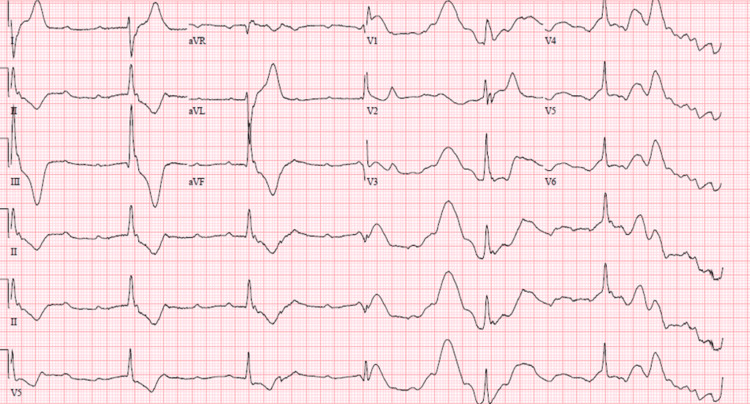
EKG revealed a third-degree heart block.

On examination, he was hemodynamically stable on a BiPAP with an oxygen requirement of 50%. Rhonchi were audible on pulmonary auscultation, his abdomen was tense, and shifting dullness was appreciated. His initial labs revealed an acute kidney injury, leukocytosis, and troponinemia. Other serum results can be found in Table 1. Imaging results included a chest radiograph showing bilateral mild-to-moderate pleural effusions, and abdominal ultrasound revealed moderate to large volume ascites. His EKG on admission revealed normal sinus rhythm with no obvious T-wave abnormalities. Due to his kidney injury, he was not able to receive any contrast. A TTE performed on admission revealed an LVEF of 45-50%, no wall motion abnormalities, normal RV function, mild tricuspid regurgitation, and RVSP 55 mmHg. During his hospital stay, he developed acute hypoxic respiratory failure with a drop in his blood pressure that was not responsive to fluid boluses. He was started on a continuous norepinephrine infusion and was transferred to the ICU for a COPD exacerbation induced by COVID-19 pneumonia, decompensated cirrhosis, and hepatorenal syndrome. Treatment included pulse dose steroids, remdesivir, and albumin. Despite these measures, his kidney function continued to decline, progressing into acute renal failure. He was started on continuous renal replacement therapy for his hypervolemia, uremia, and anuria. He continued to develop worsening acute hypoxic respiratory failure, ascites, and pleural effusions prompting intubation. Post intubation, he developed septic shock refractory to multiple vasopressors. Significant cardiac ectopy was noted on telemetry along with bradycardia with a heart rate between 25-45 bpm with hemodynamic instability. On telemetry review, he was noted to have a complete heart block (Figure 7). Repeat labs did not reveal any obvious electrolyte disturbances as the possible etiology of the arrhythmia. The patient’s family decided to pursue only palliative measures; hence further workup and management were not pursued. Bradycardia and hypotension progressed over several hours, culminating in death.

## Discussion

It is estimated that 19.7% of patients infected with SARS-CoV-2 will develop some extent of cardiac injury evidenced by elevated troponin [2]. Cardiac injury has been associated with an increased risk of requiring non-invasive and invasive ventilation and an increased risk of mortality when compared to patients without cardiac injury [2,4-6]. The pathogenesis of SARS-CoV-2-induced arrhythmias is not well established in literature. Current literature suggests that the underlying pathophysiology may be due to a direct vs. indirect injury [7-9]. In this case series, we have noted four patients with different comorbidities, hospital courses, and presumably normal cardiac function prior to COVID-19 who developed the same abnormal rhythm of complete heart block. The duration and consequences of atrioventricular (AV) nodal blockade were not consistent among the four cases, analogous to most pathologies involving a spectrum of diseases.

Complete AV block can be caused by increased vagal tone, cardiac conduction system disease (fibrosis), ischemic heart disease, cardiomyopathies (including sarcoidosis, hemochromatosis, or amyloidosis), infection (such as Lyme or viral myocarditis), hyperkalemia, thyroid disease, medications (including beta-blockers, calcium channel blockers, digoxin, adenosine, and antiarrhythmic medications), and surgical complications [4-6]. The development of arrhythmias in patients with COVID-19 is associated with an increase in mortality and morbidity. In the literature so far, no clear causal mechanism has been discovered for the development of arrhythmias associated with SARS-CoV-2 [1]. It is also notable that the frequency of cardiac arrhythmias correlates with the severity of COVID-19. This finding could indicate a direct perturbation of the electrical conduction system of the heart by the SARS-CoV-2 virus. It has been suggested that direct viral infiltration of cardiomyocytes through the angiotensin-converting enzyme-2 receptors and subsequent systemic inflammation may be one mechanism of cardiac injury [10-12]. Additionally, interstitial mononuclear inflammatory cell infiltrates were noted from autopsies of cardiac tissue in patients who passed away from COVID-19 [13]. Interestingly, a decrease in the lymphocyte number was found in serum; however, an increase in the activity of Th17 and CD8 cells harboring enzymes, such as perforin and granulysin, which can contribute to cardiac injury, fibrosis, and dysfunction, was noted [14]. Other histological analyses in critically ill patients have revealed myocardial interstitial edema expanding into the spaces of the cardiac muscles and evidence of small vessel vasculitis with neutrophilic, and lymphocytic obstruction in the lumen and blood vessel walls [15]. Furthermore, international post-mortem studies have revealed evidence of lymphocytic myocarditis and widespread macrophage infiltration in the myocardium [16]. These results are highly suggestive of COVID-19-mediated endothelial injury and cardiac myocyte injury. Studies have also shown that these activated endothelial cells can become a source of reactive oxygen species resulting in vasoconstriction and oxidative stress, ultimately leading to cell swelling and myocyte death as seen in histological analysis [15]. This can contribute to cardiac arrhythmias through decreased cardiac myocyte function and decreased signal propagation down myocytes.

Initially, ischemia via micro-thrombi was believed to be the major perpetrator of abnormal heart rhythms observed with COVID-19; however, the absence of troponinemia at the time of arrhythmia onset does not suggest that ischemia is the mainstay mechanism of arrhythmia development [13]. Electrolyte and metabolite abnormalities as an etiology of complete heart block were unlikely as there was no obvious electrolyte disturbance during the time of arrhythmias onset. Further, vagal involvement of the cardiac inhibitory receptors during myocardial ischemia resulting in vasodilation, bradycardia, and hypotension through the Bezold-Jarisch reflex, cannot be ruled out [17]. However, the prolonged involvement of the arrhythmia in three out of the four cases suggests an etiology that was not transient. It is notable that the heart block experienced in case 3 was transient (24 hours) and that even though his cardiac thrombus was extensive, he developed the arrhythmia and then spontaneously converted back to normal sinus rhythm. The narrow complex morphology seen in case 2 could suggest a pathology higher than the infra-hisian fibers, such as the bundle of His or AV node. The etiology of the complete heart block may be multifactorial.

The idea that an organism has a predilection for myocardial tissue is not uncommon. Some organisms are known to selectively affect the AV nodal apparatus, causing a spectrum of diseases analogous to what is seen in our cases. For example, in Lyme carditis, biopsies have found spirochetes interwoven within the cardiac matrix along with transmural lymphocytic infiltrate, which can lead to pathologies, including myocarditis, endocarditis, and AV conduction delays [18,19]. Further, viral myocarditis involving the Coxsackie B virus is known to cause dilated cardiomyopathy, sudden cardiac death, and arrhythmias, such as ventricular tachycardia and high-degree AV block. It is unfortunate that our cases did not undergo cardiac biopsies, and there is no post-mortem autopsy information. Hence, depending on the severity of the disease and host characteristics, SARS-CoV-2 could show a preference for the cardiac conduction system, yielding arrhythmias without elevated cardiac biomarkers in some cases.

## Conclusions

It is unclear if SARS-CoV-2 has a direct affinity for the cardiac conduction system and if the arrhythmias seen in COVID-19 are due to direct viral invasion, an inflammatory response, or other unaccounted-for etiologies such as micro-thrombi that may be contributing more than expected. However, this case series hopes to draw further awareness to unique arrhythmias that can be associated with COVID-19 and to inspire further interest in identifying the etiology of abnormal arrhythmias to improve diagnosis, management, morbidity, and mortality.
